# Association between migration and oral health-related quality of life: results from a nationally representative online survey

**DOI:** 10.1186/s12903-022-02337-5

**Published:** 2022-07-26

**Authors:** Ghazal Aarabi, Carolin Walther, Benedikt Kretzler, Larissa Zwar, Hans-Helmut König, André Hajek

**Affiliations:** 1 Department of Periodontics, Preventive and Restorative Dentistry, University Medical Center Hamburg-Eppendorf, Martinistr. 52, 20246 Hamburg, Germany; 2 Department of Health Economics and Health Services Research, University Medical Center Hamburg-Eppendorf, Hamburg Center for Health Economics, Hamburg, Germany

**Keywords:** Oral health-related quality of life, Migration, Oral health, Quality of life, Public health, Surveillance

## Abstract

**Purpose:**

To analyze the link between individuals with and without migration background and oral health-related quality of life (also stratified by sex).

**Methods:**

Data in this cross-sectional study were taken from a nationally representative survey (n = 3075, August/September 2021). The Oral Health Impact Profile (OHIP-G5) was used to measure oral health-related quality of life. Two-part models were calculated, adjusting for various covariates.

**Results:**

Individuals with migration background had lower oral health-related quality of life (total sample, Cohen’s d = − 0.30; in men, d =− 0.44; in women, d =− 0.22). Two-part models also revealed that the migration background was associated with a higher likelihood of OHIP-G5 scores of one or higher (total sample and in both sexes). Moreover, migration background was positively associated with the extent of oral health-related quality of life (conditional on OHIP-G5 scores of one or higher; total sample and in men). Furthermore, regressions showed that migration background was associated with lower oral health-related quality of life (total sample and in both sexes).

**Conclusions:**

Our study emphasized the link between having a migration background and lower oral health-related quality of life among both women and men. Maintaining oral health among individuals with a migration background is a key challenge. Culturally and socially sensitive actions should provide easy accessible oral health information and preventive measures in order to lower access barriers in dental care for individuals with migration background.

## Introduction

Inequalities in oral diseases can be seen in marginalized and poor groups of society [1]. Non-white race and ethnicity, low levels of education, low family income are risk factors for suffering from oral diseases (caries, tooth loss, periodontitis, need for dental prosthesis) [2]. A migration background does not per se cause disease initiation/development, but the accumulation of risk factors are especially seen in migrant groups that are often socioeconomically disadvantaged [3]. They experience a lot of different barriers to health services [4]. The healthcare system in general, but also the prevention programs and treatment costs (out-of-pockets payments) can differ fundamentally to their country of birth and people with migration background often present a lower utilization of health care services [5].

In 2010, the age-standardized prevalence of untreated caries lesion in permanent teeth was > 51% in Germany [6], the prevalence of tooth loss (having less than 9 remaining teeth) was 2.5% [7] and the prevalence for severe periodontitis in western Europe was 9.4% [8]. The consequences of chronic oral diseases are considerable: the chewing of food, and thus the first step of digestion, is impaired. Pronunciation may change, and of course such people often suffer from the aesthetic impairments. This is often accompanied by poor self-esteem, social isolation, and even feelings of loneliness [9]. Furthermore, chronic oral inflammation can either directly affect the general health via bacteria translocation or indirectly via immunometabolic alterations and alterations in the bone marrow [10]. Literature is reporting an association between periodontitis and atherosclerosis, hypertension, peripheral arterial occlusive disease, metabolic syndrome, diabetes and many more (reviewed in [11]).

The oral health-related quality of life index evaluates the oral health, the psychological well-being and utilization patterns for healthcare in individuals. Literature is reporting a different risk for oral health-related quality of life in men and women [12], and this gender differences can partly be explained by different accumulation of dental diseases (e.g. periodontitis, caries lesions and oral cancer) [13]. When compared to people with no migration background, people with migration background present significantly impaired oral health-related quality of life in Germany [14]. According to the Federal Office for Migration and Refugees (BAMF), 26% of the German population has a migration background (almost one third migrated from EU countries and another third from other European countries). Consequently, those migrant groups are not marginal groups of society. They display a quarter of the German population, but still literature addressing this problematic trend is rare [15–18], especially for Germany [14].

Therefore, the aim of the current study is to evaluate the association between individuals with and without migration background and oral health-related quality of life in adults with migration background in Germany (also stratified by sex).

## Material & methods

### Sample

Data for our current cross-sectional study were taken from a nationally representative online survey (n = 3,075; 18–70 years; living in Germany). It is worth noting that the questionnaire was exclusively available in one language (Germany; please also see the limitations section). Data collection took place from August to September 2021. An established market research company recruited participants (based on an online sample). They were included in a way that our sample corresponds to the distribution of gender, age as well as the federal state in the German adult population.

### Outcome: oral health-related quality of life

To measure oral health-related quality of life, we used the well-known Oral Health Impact Profile (OHIP-G5 [19]) which encompasses four factors [1] oral function, [2] orofacial pain, [3] appearance and [4] psychosocial impact. Favorable psychometric characteristics of the OHIP-G5 have been shown [19]. The items were as follows (in each case: 0-never, 1-hardly ever, 2-occasionally, 3-fairly often, and 4-very often): [1] Have you had difficulty chewing any foods because of problems with your teeth, mouth, dentures or jaw? [2] Have you felt that there has been less flavor in your food because of problems with your teeth, mouth, dentures or jaws? [3] Have you had painful aching in your mouth? [4] Have you felt uncomfortable about the appearance of your teeth, mouth dentures or jaws? [5] Have you had difficulty doing your usual jobs because of problems with your teeth, mouth, dentures or jaws? A sum score was computed. Thus, the OHIP-G5 ranges from 0 to 20 (higher scores reflect *lower* oral health-related quality of life). Cronbach’s alpha was 0.85 in our current study.

### Independent variables

The key independent variable was self-rated migration background (no; yes). It was explained as follows in the questionnaire: “A person has a migration background if he or she or at least one parent was not born with German citizenship”. This is a common way to quantify the migration background [20].

Based on prior research (e.g., [21–24] and also based on theoretical considerations, covariates were selected. More precisely, an association between various sociodemographic factors and oral health-related quality has been shown. For example, an association between age as well as education and oral health-related quality of life has been shown by Rebelo et al. [23]. Another study demonstrated an association between sex and oral health-related quality of life [25]. Moreover, an association between family status [26], children [27] as well as employment status [28] and oral health-related quality of life has been shown [26]. Moreover, an association between lifestyle factors (e.g., smoking status [29], alcohol intake [29] and sports activities [30]) and oral health-related quality has been shown. Additionally, an association between self-rated health as well as chronic diseases and oral health-related quality of life has been documented [31, 32]. Furthermore, an association between several sociodemographic, lifestyle as well as health-related factors and migration has been identified (e.g., [33–39]).

As covariates, we included sociodemographic factors: sex (men; women), age in years, children in the same household (two answer categories: no; yes), family status (distinguishing between: married, living together with spouse; married, not living together with spouse; divorced; single; widowed), labor force participation (full-time employed; retired; other), and level of education (with the following options: without school-leaving qualification; currently in school training/education; lower secondary school; intermediate secondary school; polytechnic secondary school; qualification for applied upper secondary school; upper secondary school). Furthermore, we included lifestyle- and health-related covariates: sports activities (distinguishing between: regularly, more than 4 h a week; regularly, 2–4 h a week; regularly, 1–2 h a week; less than one hour a week; no sports activity), smoking status (with the categories: never smoker; no, not anymore; yes, sometimes; yes, daily), alcohol intake (never; less often than 1–3 times per month; 1–3 times per month; once a week; several times per week; daily), vaccination against Covid-19 (with two options: no; yes), self-rated health (single-item ranging from 1 = very bad to 5 = very good) and chronic diseases (two option: presence of at least one chronic disease; absence of chronic diseases).

### Statistical analysis

Sample characteristics are displayed stratified by migration background. We also calculated the effect sizes (Cohen’s d). Thereafter, unadjusted and adjusted two-part models [40] were performed (first part: logit model; second part: generalized linear model with gamma distribution and log link function – considering the skewed distribution [41]) to analyze the link between migration background and oral health-related quality of life.

Two-part models are often used when there is a large proportion of values of zero (in our study: highest oral health-related quality of life). The “twopm” command was used in this study [40]. We also calculated average marginal effects (AME; due to reasons of interpretability of the results) reflecting the change in oral health-related quality of life associated with a one-unit change in the regressors.

The level of significance was determined at p < .05. In our study, we used Stata 16.1 (Stata Corp., College Station, Texas) for statistical analysis.

## Results

### Sample characteristics stratified by migration background

The average age was 45.5 years (SD:14.7 years) among individuals without a migration background and it was 37.0 years (SD:13.6 years) among individuals with a migration background. In sum, 49.6% were female among individuals without a migration background, whereas 62.4% were female among individuals with a migration background.

Bivariately, the migration status was associated with most of the other variables (except for children in the same household and sports activities). The average oral health-related quality of life was 2.1 (SD: 3.2) among individuals without a migration background, whereas it was 3.1 (SD: 4.1) among individuals with a migration background. According to Cohen’s d, individuals with migration background had lower oral health-related quality of life (total sample, d = − 0.30; in men, d=−0.44; in women, d=−0.22) (Table 1).

**Table 1:** Sample characteristics stratified by migration background (n = 3,075)

Variables	Not having a migration background	Having a migration background	p-value
	N = 2724 (100%)	N = 351 (100%)	
Sex			< 0.001
Men	1370 (50.3%)	132 (37.6%)	
Women	1351 (49.6%)	219 (62.4%)	
Diverse	3 (0.1%)	0 (0.0%)	
Age	45.5 (14.7)	37.0 (13.6)	< 0.001
Children in own household			0.39
No	1961 (72.0%)	245 (69.8%)	
Yes	763 (28.0%)	106 (30.2%)	
Marital status			< 0.05
Single / Divorced / Widowed / Married, not living together with spouse	1144 (42.0%)	169 (48.1%)	
Married, living together with spouse	1580 (58.0%)	182 (51.9%)	
Highest educational degree			< 0.001
Upper secondary school	1140 (41.9%)	186 (53.0%)	
Qualification for applied upper secondary school	278 (10.2%)	50 (14.2%)	
Polytechnic Secondary School	157 (5.8%)	11 (3.1%)	
Intermediate Secondary School	816 (30.0%)	72 (20.5%)	
Lower Secondary School	316 (11.6%)	31 (8.8%)	
Currently in school training/education	9 (0.3%)	0 (0.0%)	
Without school-leaving qualification	8 (0.3%)	1 (0.3%)	
Employment status			< 0.001
Full-time employed	1314 (48.2%)	144 (41.0%)	
Retired	475 (17.4%)	24 (6.8%)	
Other	935 (34.3%)	183 (52.1%)	
Smoking			< 0.001
Yes, daily	652 (23.9%)	64 (18.2%)	
Yes, sometimes	203 (7.5%)	48 (13.7%)	
No, not anymore	772 (28.3%)	71 (20.2%)	
Never smoker	1097 (40.3%)	168 (47.9%)	
Sports activities			0.13
No sports activity	747 (27.4%)	87 (24.8%)	
Less than one hour a week	542 (19.9%)	87 (24.8%)	
Regularly, 1–2 h a week	632 (23.2%)	82 (23.4%)	
Regularly, 2–4 h a week	416 (15.3%)	57 (16.2%)	
Regularly, more than 4 h a week	387 (14.2%)	38 (10.8%)	
Alcohol intake			< 0.05
Daily	169 (6.2%)	17 (4.8%)	
Several times per week	520 (19.1%)	44 (12.5%)	
Once a week	438 (16.1%)	57 (16.2%)	
1–3 times per month	466 (17.1%)	66 (18.8%)	
Less often	617 (22.7%)	98 (27.9%)	
Never	514 (18.9%)	69 (19.7%)	
Vaccinated against Covid-19			< 0.01
No	505 (18.5%)	88 (25.1%)	
Yes	2219 (81.5%)	263 (74.9%)	
Chronic diseases			< 0.05
Absence of chronic diseases	1542 (56.6%)	223 (63.5%)	
Presence of at least one chronic disease	1182 (43.4%)	128 (36.5%)	
Self-rated health (1 = very bad to 5 = very good)	3.6 (0.9)	3.7 (0.8)	< 0.01
Oral health-related quality of life (OHIP-G5; from 0 to 20, with higher scores indicating lower oral health-related quality of life)	2.1 (3.2)	3.1 (4.1)	< 0.001

Independent t-tests or Chi²-tests were conducted, as appropriate

A description of the OHIP-G5 items (also referring to the four dimensions) is displayed in Table 2.

**Table 2:** Description of the OHIP-G5 among the total sample

OHIP-G5 items	Mean (SD) / n (%)
Difficulty chewing any foods (indicator of Oral Function): Mean (SD), from 0 = never to 4 = very often	0.5 (0.9)
Never	2126 (69.1%)
Hardly ever	488 (15.9%)
Occassionally	325 (10.6%)
Fairly often	93 (3.0%)
Very often	43 (1.4%)
Felt less flavor in food (indicator (indicator of Oral Function): Mean (SD), from 0 = never to 4 = very often	0.4 (0.8)
Never	2329 (75.7%)
Hardly ever	428 (13.9%)
Occassionally	243 (7.9%)
Fairly often	59 (1.9%)
Very often	16 (0.5%)
Painful aching in your mouth (indicator of Orofacial Pain): Mean (SD), from 0 = never to 4 = very often	0.5 (0.9)
Never	2256 (73.4%)
Hardly ever	376 (12.2%)
Occassionally	336 (10.9%)
Fairly often	70 (2.3%)
Very often	37 (1.2%)
Felt uncomfortable about the appearance (indicator of Orofacial Appearance): Mean (SD), from 0 = never to 4 = very often	0.6 (1.0)
Never	2100 (68.3%)
Hardly ever	400 (13.0%)
Occassionally	369 (12.0%)
Fairly often	135 (4.4%)
Very often	71 (2.3%)
Difficulty doing your usual jobs (indicator of Psychosocial impact): Mean (SD), from 0 = never to 4 = very often	0.3 (0.7)
Never	2605 (84.7%)
Hardly ever	247 (8.0%)
Occassionally	145 (4.7%)
Fairly often	53 (1.7%)
Very often	25 (0.8%)

### Regression analysis

Findings of two-part models are shown in Table 3.

**Table 3:** Determinants of oral health-related quality of life. Two-part models (unadjusted models; (1) Logit (2) GLM^1^)

	Total sample			Women			Men		
Independent variables	Logit OR (95% CI)	GLM b (95% CI )	Predict. margin (95% CI)	Logit OR (95% CI )	GLM b (95% CI )	Predict. margin (95% CI)	Logit OR (95% CI )	GLM b (95% CI )	Predict. margin (95% CI)
Migration background (Ref.: No migration background)	1.54*** (1.23–1.94)	0.19** (0.07–0.30)	0.88*** (0.53–1.24)	1.42* (1.06–1.89)	0.13+ (− 0.02-0.29)	0.64** (0.19–1.09)	1.70** (1.18–2.44)	0.29*** (0.12–0.46)	1.28*** (0.70–1.86)
Observations	3075	3075	3075	1564	1564	1564	1502	1502	1502

Two-part models with oral health-related quality of life as outcome measure; ^1^ Generalized linear model (GLM) with log link and gamma distribution; OR = odds ratio; 95% CI in parentheses; *** p < .001, ** p < .01, * p < .05, + p < .10

In unadjusted two-part models, the migration background was associated with a higher likelihood of OHIP-G5 scores of one or higher (first part: logit model) in the total sample (OR: 1.54, p < .001) and in both sexes (women, OR: 1.42, p < .05; men, OR: 1.70, p < .01). In addition, migration background was positively associated with the extent of oral health-related quality of life (conditional on OHIP-G5 scores of one or higher; second part) in the total sample (b = 0.19, p < .01) and in men (b = 0.29, p < .001). Furthermore, following the AME, migration background was associated with lower oral health-related quality of life in the total sample (AME: 0.88, p < .001) and in both sexes (women, AME: 0.64, p < .01; men, AME:1.28, p < .001) (Table 3).

Two-part models (adjusted models) also revealed that the migration background was associated with a higher probability of OHIP-G5 scores of one or higher (first part: logit model) in the total sample (OR: 1.60, p < .001) and in both sexes (women, OR :1.57, p < .01; men, OR: 1.69, p < .01). Moreover, migration background was positively associated with the extent of oral health-related quality of life (conditional on OHIP-G5 scores of one or higher; second part) in the total sample (b = 0.17, p < .01) and in men (b = 0.19, p < .05). Additionally, according to the AME, migration background was associated with lower oral health-related quality of life in the total sample (AME: 0.84, p < .001) and in both sexes (women, AME: 0.65, p < .01; men, AME: 0.98, p < .001) (Table 4). The interaction terms (migration x sex) did not achieve statistical significance in these models (interaction term; first part: OR: 0.87, 95% CI: 0.53 − 1.40, p = .56; second part, b = −0.14, 95% CI:− 0.37-0.09, p = .23).

**Table 4:** Determinants of oral health-related quality of life. Two-part models (adjusted models; (1) Logit (2) GLM^1^)

	Total sample			Women			Men		
Independent variables	Logit OR (95% CI)	GLM b (95% CI )	Predict. margin (95% CI)	Logit OR (95% CI )	GLM b (95% CI )	Predict. margin (95% CI)	Logit OR (95% CI )	GLM b (95% CI )	Predict. margin (95% CI)
Migration background (Ref.: No migration background)	1.60*** (1.25–2.03)	0.17** (0.06–0.29)	0.84*** (0.49–1.19)	1.57** (1.15–2.15)	0.11 (− 0.04-0.27)	0.65** (0.22–1.08)	1.69** (1.14–2.51)	0.19* (0.01–0.37)	0.98*** (0.40–1.55)
Potential confounders	✓	✓	✓	✓	✓	✓	✓	✓	✓
Observations	3075	3075	3075	1564	1564	1564	1502	1502	1502

Two-part models with oral health-related quality of life as outcome measure; ^1^ Generalized linear model (GLM) with log link and gamma distribution; OR = odds ratio; 95% CI in parentheses; *** p < .001, ** p < .01, * p < .05, + p < .10; Potential confounders include sex (only in total sample), age, marital status, education, presence of children in the same household, smoking status, alcohol intake, sports activities, vaccinated against Covid-19, presence of chronic diseases and self-rated health

## Discussion

### Main findings

The aim of this study was to investigate the association between migration background and oral health-related quality of life (also stratified by sex). Our study showed small (women) to medium (men) differences in oral health-related quality of life (in terms of effect size) between individuals with migration background and their counterparts. Two-part models revealed that the migration background was associated with a higher likelihood of OHIP-G5 scores of one or higher total sample and in both sexes. Moreover, migration background was positively associated with the extent of oral health-related quality of life (total sample and in men). Furthermore, migration background was associated with lower oral health-related quality of life (total sample and in both sexes).

### Previous research and possible explanations

The Platform of Longitudinal Studies on Immigrant Families (PELFI) evaluated data, in a cross-sectional study design, from 401 adults aged over 18 years from Spain, Morocco, Colombia, and Ecuador [16]. The study population consisted of middle-aged adults (Males: 48.4% between 45 and 54 years; Females: 36.2% between 45 and 54 years), when compared to our data with a mean age of the study population of 37.0 years (SD 13.6). The authors applied the OHIP 14 instrument and revealed a statistically significant association between immigration and OHRQoL only for Moroccan women (OR:5.08; 95% CI:1.93–13.34). We could detect a significant association between migration background and lower oral health-related quality of life for men and women, which is in line with another U.S. American study. The authors included data from 733 subjects from the TEETH (“Trials to Enhance Elders’ Teeth and Oral Health”) study. The distribution of men and women was almost homogeneous (44.4% males) and the authors reported a significant association between ethnicity (foreign-born vs. North American) and number of years after immigrating to North America with oral health-related quality of life (assessed via the Geriatric Oral Health Assessment Index (GOHAI)) [15].

Unfortunately, a more detailed comparison of OHRQoL Indices in adult migrant populations in Germany is still difficult due to a lack of studies. We could only identify one German study assessing oral health-related quality of life. However, this study focused on children/adolescents and it is therefore difficult to compare with our study [14].

The lower OHRQoL in people with and without migration background can have several reasons: Migration background is strongly associated with adverse oral health outcomes including a higher prevalence of untreated caries, severe periodontitis, and a higher number of missing teeth, which are all related to low oral health-related quality of life. The poor oral health of people with migration background relates to a lower socio-economic status (SES) [42] [43], high intake of sugar-rich beverages (i.e. sweetened tea), low oral health literacy [44], low and more pain than prevention oriented utilization of dental care services, poor German language skills, and other lifestyle and cultural difference in comparison to the non-migrant population [45]. In addition, people with migration background often live in socially deprived areas with a low physician-population ratio [46, 47].

### Strengths and limitations

One strength is the large sample which matches the distribution of sex, age group and federal state in the German adult population. Two-part models were used. It was adjusted for various covariates in regression analysis. The OHIP-G5 was used to quantify oral health-related quality of life. It is highly associated with longer versions (e.g., version with 49 items: r = .93) [48]. A limitation is that the questionnaire was only available in German language. Thus, individuals with a migration background and with insufficient German language skills may be excluded. We therefore assume that the actual differences in oral health-related quality of life between the two groups may be underestimated. Moreover, future research is required which distinguishes migration status in further detail (e.g., first-generation migrants and second-generation migrants). The current study has a cross-sectional design, with the acknowledged limitations.

## Conclusions

Our study emphasized the association of having a migration background and lower oral health-related quality of life among both women and men. Maintaining oral health among individuals with a migration background is a key challenge. Culturally and socially sensitive actions should provide easy accessible oral health information and preventive measures in order to lower access barriers in dental care for individuals with migration background.

## Data Availability

The datasets generated and/or analysed during the current study are not publicly available due to legal restrictions, but are available from the corresponding author on reasonable request.
